# Supplementary material for: Identification of Freezing-Responsive microRNAs and Their Targets in Chinese Jujube by Small RNA and Degradome Sequencing
Source: Int J Mol Sci. 2026 Sep 4;27(17):7912. doi: 10.3390/ijms27177912 (PMC13566890; doi:10.3390/ijms27177912)
Supplement: Supplementary file 1 [file ijms-27-07912-s001.zip › Supplementary Material S2/novel_miR_217.pdf]

Provisional ID : novel_miR_217
Score total : 0.4
Score for star read(s) : -1.3
Score for read counts : 0
Score for mfe : 0.1
Score for randfold : 1.6
Score for cons. seed :
Total read count : 343
Mature read count : 343
Loop read count : 0
Star read count : 0

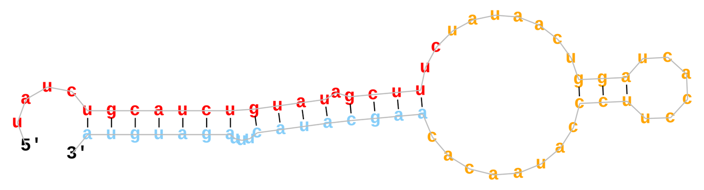

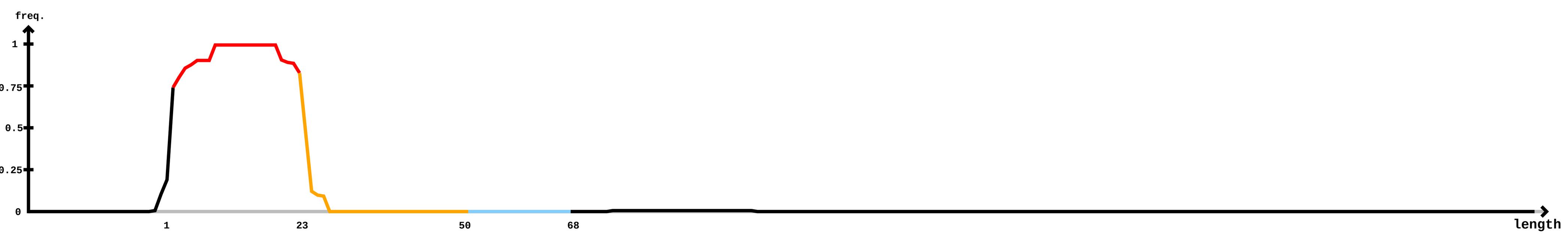

Mature Star

```
5'- cuacuaucaaggucuuuagucauaucugcaucuguauagcuuucuauaacuggaucaccuucccauaacacaagcauacuuuagauguacuuuuaagauacuugagaauuuacuugacagcuuuccaguguucuuuuccaaaauuugaaagaaguauacuuauuguaccaauugcaugagcaauauuugguuuaguaaacaguauugcaugcacuuuccaccacuaaagaauaaggcacugaagcuaucu -3'
    ......((((((((((.(((.(((((((.....((((.((((.........(((......))).......))))))))..))))))))))...))))).)))))..........((.(((((..((((((.((((((........(((((..(((((.....)))))....(((((..((((((((..((((...)))))))))))))))))))))))).........))).))))))))))))))).)).
```

	exp reads	mm	sample
..................Acauaucugcaucuguauagc	1	1	JCK-1
..................ucauaucugcaucuguauagc	1	0	JCK-3
...................cauaucugcaucuguauagc	7	0	JCK-3
...................cauaucugcaucuguauagc	3	0	LT-2
...................cauaucugcaucuguauagc	9	0	JCK-2
...................cauaucugcaucuguauagc	3	0	LT-3
...................cauaucugcaucuguauagc	1	0	LT-1
...................cauaucugcaucuguauagc	4	0	JCK-1
...................cauaucugcaucuguauagcu	1	0	LT-1
...................cauaucugcaucuguauagcu	2	0	LT-2
...................cauaucugcaucuguauagcu	1	0	JCK-2
...................cauaucugcaucuguauagcuuuc	3	0	LCK-3
....................auaucugcaucuguauagcu	1	0	LT-3
....................auaucugcaucuguauagcuuu	3	0	JCK-2
....................auaucugcaucuguauagcuuu	4	0	JCK-1
....................aNaucugcaucuguauagcuuu	1	1	JCK-1
....................auaucugcaucuguauagcuuu	1	0	LT-1
....................auaucugcaucuguauagcuuu	2	0	LT-2
....................auaucugcaucuguauagcuuu	4	0	JCK-3
....................auaucugcaucuguauagcuuuc	3	0	JCK-1
....................Cuaucugcaucuguauagcuuuc	1	1	JCK-1
....................auaucugcaucuguauagcuuuc	5	0	JCK-2
....................auaucugcaucuguauagcuuuc	1	0	LT-2
....................auaucugcaucuguauagcuuucu	1	0	JCK-3
....................auaucugcaucuguauagcuuucu	1	0	JCK-1
....................auaucugcaucuguauagcuuucu	1	0	JCK-2
....................auaucugcCucuguauagcuuucu	1	1	JCK-2
.....................uaucugcaucuguauagc	1	0	LT-3
.....................uaucugcaucuguauagc	1	0	LT-1
.....................uaucugcaucuguauagcuu	2	0	LT-1
.....................uaucugcaucuguauagcuuu	2	0	JCK-2
.....................uaucugcaucuguauagcuuuc	12	0	LT-2
.....................uaucugcaucuguauagcuuuc	29	0	JCK-1
.....................uaucugcaucuguauagcuuuc	63	0	JCK-2

Mature **Star**

cuacuaucaaggucuuuaguca**uaucugcaucuguauagcuuuc**uauaacuggaucaccuucccauaacac**aagcauacuuuagaugua**cuuuuaagauacuugagaauuuacuugacagcuuuccaguguucuuuuccaaaauuugaaagaaguauacuuauuguaccaauugcaugagcaauauuugguuuaguaaacaguauugcaugcacuuuccaccacuaaagaauaaggcacugaagcuaucu

uaucugcaucuguauagcuuuc	46	0	JCK-3
uaucugcaucuguauCgcuuuc	1	1	JCK-2
uaucugcaucuguauagcuuuc	16	0	LT-3
uaucugcaucuguauagcuuuc	13	0	LT-1
uaucugcaucuguauagcuuucu	1	0	JCK-2
uaucugcaucuguauagcuuucu	1	0	JCK-3
uaucugcaucuguauagcuuucua	1	0	JCK-3
uaucugcaucuguauagcuuucua	2	0	JCK-2
aucugcaucuguauagcuuu	3	0	LT-2
aucugcaucuguauagcuuuc	1	0	JCK-3
aucugcaucuguauagcuuuc	5	0	JCK-1
aucugcaucuguauagcuuuc	2	0	LT-3
aucugcaucuguauagcuuuc	3	0	LT-1
aucugcaucuguauagcuuuc	7	0	JCK-2
ucugcaucuguauagcuuuU	1	1	LT-3
ucugcaucuguauagcuuuc	5	0	JT-3
ucugcaucuguauagcuuuc	4	0	JT-2
ucugcaucuguauagcuuuc	1	0	JCK-2
ucugcaucuguauagcuuuc	2	0	LT-1
ucugcaucuguauagcuuuc	1	0	JCK-3
ucugcaucuguauagcuuucu	1	0	LT-1
ucugcaucuguauagcuuucua	4	0	JCK-2
cugcaucuguauagcuuuc	2	0	LT-2
cugcaucuguauagcuuuU	2	1	LT-2
cugcaucuguauagcuuucu	1	0	JCK-1
cugcaucuguauagcuuucuaC	1	1	JCK-2
cugcaucuguauagcuuuAuau	1	1	JCK-3
ugcaucuguauagcuuuA	2	1	LT-2
ugcaucuguauagcuuuc	2	0	LT-1
ugcauUuguauagcuuucu	3	1	LT-2
ugcaucuguauagcuuucu	1	0	LT-1
ugcaucuguauagcuuucua	1	0	LT-3
Gucuguauagcuuucuaua	14	1	LCK-2
Gucuguauagcuuucuaua	18	1	LCK-1
agauacuugagaauuuacuugaca	1	0	JCK-3
agauacuugagaauuuacuugaca	1	0	JCK-1
